# A Network-Based Approach to Glioma Surgery: Insights from Functional Neurosurgery

**DOI:** 10.3390/cancers13236127

**Published:** 2021-12-05

**Authors:** Nardin Samuel, Artur Vetkas, Aditya Pancholi, Can Sarica, Aaron Loh, Jurgen Germann, Irene E. Harmsen, Jordy Tasserie, Vanessa Milano, Kazuaki Yamamoto, Suneil K. Kalia, Paul N. Kongkham, Andres M. Lozano

**Affiliations:** 1 Division of Neurosurgery, Toronto Western Hospital, University Health Network, Toronto, ON M5G 2C4, Canada; nardin.samuel@mail.utoronto.ca (N.S.); artur.vetkas@uhnresearch.ca (A.V.); adityap227@gmail.com (A.P.); can.sarica@uhn.ca (C.S.); aaron.loh@uhnresearch.ca (A.L.); germannj@gmail.com (J.G.); irene.harmsen@uhn.ca (I.E.H.); jordy.tasserie@uhnresearch.ca (J.T.); vmilano@alumni.nd.edu (V.M.); kazuaki.yamamoto@uhnresearch.ca (K.Y.); suneil.kalia@uhn.ca (S.K.K.); paul.kongkham@uhn.ca (P.N.K.); 2 Krembil Research Institute, Toronto, ON M5T 2S8, Canada; 3 Department of Neurology and Neurosurgery, University of Tartu, 50090 Tartu, Estonia; 4 MD/PhD Program, Faculty of Medicine, University of Toronto, Toronto, ON M5S 1A4, Canada; 5 Toronto Rehabilitation Institute (KITE), University Health Network, University of Toronto, Toronto, ON M5G 2C4, Canada; 6 Centre for Advancing Neurotechnological Innovation to Application (CRANIA), University of Toronto, Toronto, ON M5S 1A4, Canada

**Keywords:** glioma, connectivity, networks, functional neurosurgery, neuroplasticity

## Abstract

**Simple Summary:**

This manuscript details the literature and discussion around revolutionizing the neurosurgeon’s approach to surgery for brain tumors by conceptualizing these tumors as entities within functional networks. We hope that the work detailed herein will aid in establishing neurosurgical paradigms to optimize planning for brain tumor surgery to improve functional outcomes for all patients.

**Abstract:**

The evaluation and manipulation of structural and functional networks, which has been integral to advancing functional neurosurgery, is beginning to transcend classical subspecialty boundaries. Notably, its application in neuro-oncologic surgery has stimulated an exciting paradigm shift from the traditional localizationist approach, which is lacking in nuance and optimization. This manuscript reviews the existing literature and explores how structural and functional connectivity analyses have been leveraged to revolutionize and individualize pre-operative tumor evaluation and surgical planning. We describe how this novel approach may improve cognitive and neurologic preservation after surgery and attenuate tumor spread. Furthermore, we demonstrate how connectivity analysis combined with neuromodulation techniques can be employed to induce post-operative neuroplasticity and personalize neurorehabilitation. While the landscape of functional neuro-oncology is still evolving and requires further study to encourage more widespread adoption, this functional approach can transform the practice of neuro-oncologic surgery and improve the care and outcomes of patients with intra-axial tumors.

## 1. Introduction

The interaction between glial tumors and structural and functional neuronal networks is becoming increasingly recognized and is reshaping our understanding of the impact of these infiltrative lesions on global brain function. Gliomas adopt mechanisms to promote progression through newly formed neuroglioma synapses that are thought to impact the electrical activity and function of existing neural pathways [1,2]. In parallel, an understanding of the overall structural and functional connectivity of the brain has emerged, alongside an awareness of the neurologic and neuropsychiatric effects that intra-axial mass lesions can have on neural networks [3]. As such, neurosurgical oncology strategies are increasingly taking into consideration updated models of the anatomical–functional architecture of the brain [4]. Conventional topographic- and tumor-oriented surgical planning is now evolving to encompass network-oriented surgery, tailored to the global structural and functional profile of each individual patient [5,6]. Innovations in neuroimaging and connectivity analyses are driving whole-brain network approaches to neurosciences in general, and functional neurosurgery in particular [7]. For example, the field of deep brain stimulation (DBS) surgery is progressing away from pre-operative identification of focal regions of stimulation and towards modulating distributed brain networks [8,9,10]. Similar views have been adopted in epilepsy surgery, where network analysis techniques refine surgical planning [11,12]. 

This paradigm shift of network analyses in functional neurosurgery can be applied to neuro-oncologic surgery to serve three important goals: (1) Mitigating tumor spread by considering the invasive nature of gliomas and their growth along white matter tracts, or so-called ‘oncologic disconnection’ [6,13]; (2) Preserving critical networks to maintain cognitive, social and occupational function post-operatively through recognition of the impact of resections on neural circuitry [6]; (3a) Harnessing neuroplasticity to induce functional reorganization through targeted neuromodulation, allowing for extended tumor resections [14]; and, (3b) Personalized strategies for post-operative neurorehabilitation through neuromodulation guided by connectivity maps.

There are numerous parallels between the functional neurosurgery paradigms centered on neural networks and those needed in neuro-oncologic surgery. Drawing upon network-based principles in functional neurosurgery, including DBS and non-invasive neuromodulation, we detail the potential role of connectivity analyses in improving oncologic and functional outcomes. As we will describe herein, a network-based approach to glioma surgery can improve the neurosurgical management of patients and transform the traditional lesion-oriented approach of neuro-oncologic surgery into functionally tailored resections, aimed at optimizing both oncologic and global functional outcomes. 

## 2. What Is Connectomics?

### Overview of Connectomic Methodologies and Applications

The term connectomics broadly refers to the study of networks of structurally and functionally connected regions within the central nervous system. Connectivity can be measured and inferred using both neuroimaging and neurophysiological methods such as diffusion tensor imaging (DTI), functional MRI (fMRI), electroencephalography (EEG), magnetoencephalography (MEG), electrocorticography (ECoG), and awake brain mapping [15]. Such studies have yielded novel insights into brain regions traditionally regarded as ‘non-eloquent’ that may actually be essential for brain function, including anatomical regions involved in mentalizing, semantic processing, and language expression [4]. In addition, individual connectomic analyses have expanded our understanding of anatomical–functional correlation, including the identification of motor speech areas outside of the traditional topographic location of Broca’s area, characterization of the medial frontal cognitive control networks, and establishment of the second ventral stream of language processing [16]. Structural connectivity is typically based on tractography (e.g., DTI) and provides an estimation of axonal fiber or tract connections between topographic brain regions [15,17]. Functional connectivity can be assessed using various aforementioned modalities, including fMRI, EEG, and MEG. While structural connectomes provide organic pathways for neuronal activity, functional connectomes may inform indirect connections, multiple inputs, or synaptic changes [18]. Therefore, both structural and functional connectomics are informative and complementary approaches to better resolve our understanding of brain connectivity. Although discordance between modalities may be observed, it is important to consider that each method is governed by specific principles and should not be interpreted as the failure of an individual method [15]. Information gathered from awake neuro-oncological, epilepsy, and DBS surgeries is used to reinforce radiological and neurophysiological models of brain networks. Network analysis can be used to better understand the structural and functional connections linking distinct brain areas in general, and in the context of an intra-axial lesion in particular. 

**Figure 1:**
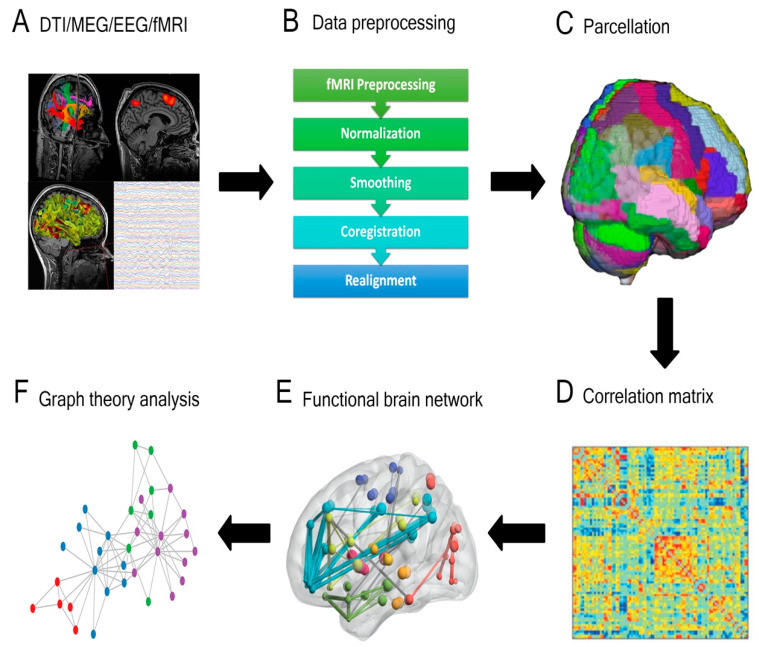
General overview of a connectivity analysis pipeline. (**A**) Structural or functional data are acquired and (**B**) pre-processed, then (**C**) parcellated by dividing the brain into distinct regions. A (**D**) correlation matrix is then created to estimate the connectedness between regions and (**E**) functional brain networks are generated. (**F**) Graph theory analysis is applied to delineate nodes, edges, and central hubs. (DTI = diffusion tensor imaging; MEG = magnetoencephalography; EEG = electroencephalography; Fmri = functional magnetic resonance imaging).

Assembling a connectome using any of the aforementioned approaches utilizes an approximately similar pipeline (Figure 1). The brain is first split into distinct regions through a process known as parcellation. In the case of functional connectomic methodologies, such as fMRI, a blood-oxygen-level-dependent (BOLD) time series is extracted from each parcel and compared with the temporal data from the remaining parcels [15]. In contrast, generating a structural connectome involves applying each parcel as a seed within the tractography iteration and the number of fibers subsequently informs putative connections between regions [15]. Through either approach, connectivity between distinct brain areas is quantified, often illustrated as a connectivity matrix. This can then be further processed using techniques such as graph theory, whereby specific regions (nodes or hubs) and the links between these regions (edges) are studied [19,20]. 

## 3. Integrating Connectomic Analysis into the Glioma Peri-Operative Pipeline: Lessons from Functional Neurosurgery

### Demonstrated Utility of Connectomics within Functional Neurosurgery

Connectivity studies have been successfully applied to various indications within functional neurosurgery. For example, the field of DBS is increasingly conceptualizing DBS as a tool to modulate brain networks [21,22]. DBS offers a window of insight into brain function associated with cognition and emotions, as do awake tumor resections with brain mapping. Viewing DBS as a modality to interrogate dysfunctional neural circuits, or ‘circuitopathies’, has led to broader applications of DBS [23]. In particular, DBS is successfully being used to target mood and cognitive circuits for psychiatric and Alzheimer’s disease, respectively [23]. Similarly, pre-operative connectomic studies using DTI, fMRI, and resting-state MEG have shown clinical utility in characterizing patients who have a higher likelihood of responding to vagus nerve stimulation (VNS) for intractable epilepsy and in DBS parameter selection for Parkinson’s disease (PD) [24,25]. Moreover, the efficacy of subthalamic nucleus (STN) DBS for PD has been associated with a distinct structural and functional connectivity profile [26]. The efficacy of DBS and VNS is, in part, due to the modulation of remote brain regions that communicate with the site of stimulation and reorganization of neural networks [27,28,29]. 

Recognizing these remote effects of DBS, one can also conceptualize that connectomic analysis may provide insight into the underlying basis of adverse effects or undesirable symptoms occurring secondary to DBS. Such an approach has been used to map the underlying connectivity of DBS-associated flashback phenomena following forniceal DBS, panic attacks induced by inferior thalamic peduncle DBS, and seizures following subcallosal cingulate DBS for refractory anorexia nervosa [30,31,32]. Similarly, connectivity-derived models can guide effective DBS for less well-understood pathologies, such as central post-stroke pain and neuropsychiatric indications, including obsessive-compulsive disorder [8,33]. Similar analyses were undertaken to investigate the perturbed networks leading to post-operative morbidity in glioma surgery. One study employed lesion-network mapping (LNM) to examine the connectomic basis for post-operative depression in a patient with a cingulate diffuse low-grade glioma [34]. This analysis drew upon principles of connectomic analysis and functional neurosurgery to characterize the neuroanatomical basis of this post-operative morbidity. Specifically, networks associated with subgenual cingulate DBS were compared to networks affected both by the tumor and surgical approach. The analysis showed that the surgical corridor had greater overlap with DBS-based depression networks [34]. 

Collectively, there is an emerging body of literature supporting the feasibility and clinical utility of brain mapping in individual patients, and this can broadly be translated to glioma patients. Although integrating these studies into a pre-surgical planning scheme may present challenges in implementation and application, such analyses hold the potential to identify brain regions that are essential to network function, which may otherwise be at risk from tumor resection [20].

## 4. Practical Applications of Connectivity for Glioma Surgery

### 4.1. Conceptualizing Glioma Resection in Terms of Oncologic Disconnection

The primary goals of integrating connectivity into surgical planning include (1) sparing of functional networks leading to anticipated decreases in post-operative morbidity and (2) so-called ‘oncologic’ disconnection. The latter can broadly refer to disconnection of pathways of tumor spread but may also refer to resection of seizure onset zone and disconnection of epileptogenic networks. Proving the possibility of using pre-operative connectivity studies for the sparing of functional networks, one study of glioblastoma patients demonstrated that ‘connectomic signatures’ derived from fMRI connectomes could identify regions critical to network function [20]. However, intra-operative direct cortical and subcortical stimulation remains the gold standard for mapping.

### 4.2. Preservation of Cognitive Eloquence, Higher-Order Behavioral and Social Functions

**Figure 2:**
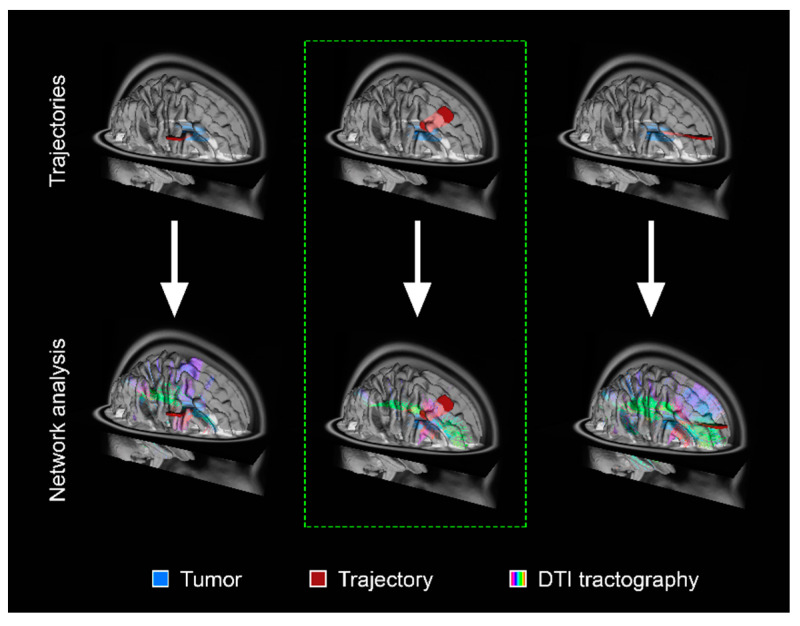
Schematic of pre-operative structural connectivity to identify optimal trajectories for tumor resection and minimal network disruption. The top panels demonstrate a representative schematic of an intra-axial tumor (blue) and possible surgical trajectories (red). The bottom panels show the corresponding network analysis based on DTI tractography. The trajectory demarcated by the dashed green line represents the surgical trajectory with minimal structural network disruption. A similar approach can be applied to visual representations of functional networks derived from other modalities to infer connectivity.

A global perspective that considers the impact of tumors on neural circuitry can endeavor to preserve critical networks to maintain cognitive, social, and occupational function post-operatively [6]. Therefore, such strategies must be coupled with formal neuropsychiatric assessments in both the pre-operative and post-operative phases. The concept of cognitive eloquence in neurosurgery has been proposed whereby cortical and subcortical regions of the brain, which are not known to have a definite neurological function, may result in cognitive morbidity when traversed surgically [35]. Functional MRI and/or DTI acquired pre-operatively could be leveraged to capture the patient-specific connectome and delineate specific cognitive or affective networks [36]. Such an approach can therefore allow the surgeon to plan a minimally disruptive trajectory (Figure 2).

This is clinically relevant as earlier detection of gliomas had led to the increasing recognition of preserving quality of life for these patients [37]. Potential regions of cognitive eloquence are thought to coincide with areas central in interconnected brain networks [35,38]. Evidence for the existence of these so-called brain ‘hubs’ is derived from several studies, including network analysis of DTI data as well as resting-state MRI [39,40]. Commonly identified hubs, or areas of centrality, include the dorsal superior prefrontal cortex, non-dominant medial superior frontal gyrus, anterior insula, temporal–occipital cortex, precuneus, and the superior and medial occipital gyri [38]. In addition to defining potential regions of functional and cognitive eloquence, it is important to have an understanding of neuroplasticity in glioma surgery since functional networks can undergo marked reorganization in the face of an infiltrative or compressive mass lesion [41]. Functionally, these may result in a shift of ‘hubs’ from their location in the normal brain due to neuroplastic changes imposed by the presence of an infiltrative mass lesion.

## 5. Employing Neuromodulation Strategies in Glioma Surgery to Influence Peri-Operative Functional Reorganization and Promote Post-Operative Neurorehabilitation

**Figure 3:**
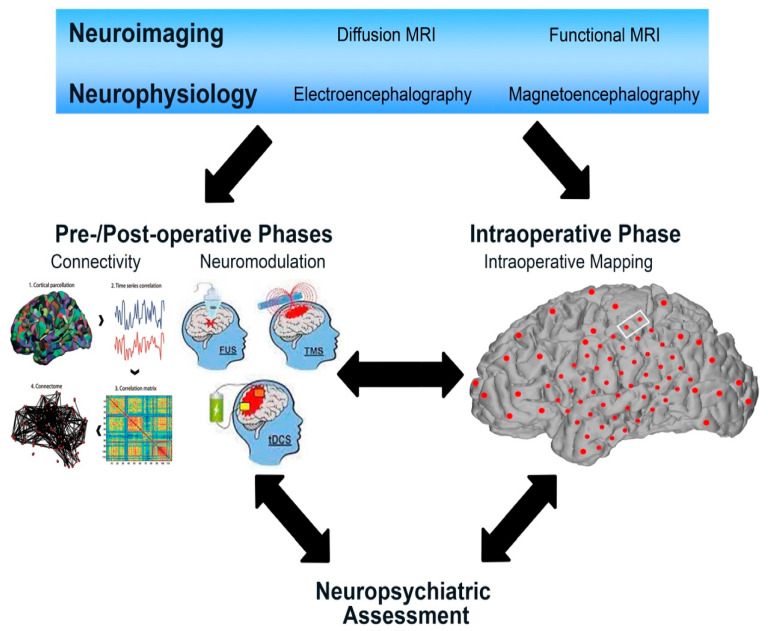
Visual representation of the integration of connectivity into the neurosurgical care management of glioma. Pre-operative investigations, including neuroimaging and neurophysiology, can be used to derive pre- and post-operative connectivity analyses and can also serve to inform neuromodulatory strategies for pre-habilitation or post-operative neurorehabilitation. Intraoperative mapping can be used as an adjunct mapping tool, and the functions assessed can be derived from connectivity analyses. Neuropsychiatric assessment can occur simultaneously to validate and assess cognitive and behavioral functions and correlate these with connectivity.

Utilizing connectomics in the peri-operative pipeline affords strong potential for the use of network mapping to expand our knowledge of the mechanisms of pre- and post-resection functional compensation and to conceptualize neuromodulation strategies to promote post-operative neurorehabilitation (Figure 3). 

These methods can potentially be used pre-operatively to facilitate plasticity in regions infiltrated by glioma. We anticipate that network-based analyses can inform neuromodulatory strategies to improve cognitive and behavioral outcomes by modulating physiological pathways. While the concept of using neuromodulation to target distributed brain networks is not new, there is novelty in our ability to visualize networks, correlate them with symptoms, and utilize this information to personalize strategies to improve functional outcomes [42]. 

### 5.1. Harnessing the Neuroplastic Potential of the Brain to Modulate Function

Neuroplasticity is an intrinsic property of neural pathways that enables the formation and consolidation of new pathways by refining existing connections, pruning or promoting neurogenesis, and adding new synaptic connections [43]. This can be broadly conceptualized as a balanced interplay of mechanisms promoting change and those promoting the stability of neural networks [44]. With this understanding of neuroplasticity and emphasis on functional outcomes informed by connectivity studies, there is a putative role for neuromodulation of neural networks in both peri-operative reshaping and post-operative neurorehabilitation. The highest potential for plasticity occurs at the cortical level, while subcortical plasticity is less robust [41]. Studies of patients with ischemic stroke have shown that damage of white matter pathways leads to worse neurologic outcomes than lesions of the cortex [41]. This implies that eloquent axonal connectivity should be preserved to enable post-lesional compensation. Non-invasive brain stimulation (NIBS) methods, such as transcranial magnetic stimulation (TMS), low intensity focused ultrasound, and transcranial direct current stimulation (tDCS), can elicit changes in cortical excitability and potentiate plasticity following brain insult. This may potentially represent an important role for neuromodulation in promoting peri-operative reshaping and post-operative functional recovery [6]. However, as our understanding of brain circuits facilitating plasticity evolves, it remains to be determined whether ‘pre-habilitation’ strategies will be more effective than post-resection rehabilitation. Further studies are also needed to investigate the impact of pre-habilitation on functional outcomes.

Functional studies using fMRI, repetitive TMS (rTMS), and MEG of patients pre- and post-resection of low-grade gliomas have shown that cortical functional reorganization frequently occurs [45,46,47,48,49]. Several studies have identified neuroplasticity in the context of slow-growing lesions such as low-grade gliomas and provide support for a model by which staged surgery may be favorable in this clinical context to drive plasticity of subcortical pathways [50,51]. Evidence of remodeling in patients with gliomas has been demonstrated through resection of canonically ‘eloquent’ regions with preservation of function [52]. In the case of DBS, although the mechanisms of deep brain stimulation are currently under investigation, studies have shown that the efficacy of STN DBS in patients with PD is at least in part due to restoration of cortical plasticity [53]. 

MRI-guided focused ultrasound (MRgFUS) is being used as a neuromodulatory strategy for various central nervous system pathologies and may have a future role in the neuro-oncology setting [45]. In particular, MRgFUS may be utilized to stimulate specific brain regions and pathways in order to investigate its potential application in targeted rehabilitation. Moreover, MRgFUS is also being used as a tool to open the blood–brain barrier transiently, and this may hold promise in improving adjunct therapy such as drug delivery to glioma patients [54,55].

### 5.2. Potential Neuromodulatory Strategies for Further Study

With the evolving knowledge of brain neuroplasticity, it is rational to envision a treatment strategy for lower-grade glioma patients whereby neuroplasticity is induced by way of cortical stimulation and followed by surgery to resect the lesion. Importantly, neuroplasticity in patients with glioma occurs in functional areas, including the precentral gyrus and regions governing language [56,57]. However, these areas have a relatively lower index for functional compensation in comparison to speech areas, and as such, stimulation can provide a sensible approach to promote neuroplastic mechanisms in a functional–oncologic staged fashion [58].

Cortical stimulation has been shown to suppress function in the stimulated region and, through this training, promote plastic changes in neural circuitry [59]. Analyses of patients with brain tumors in eloquent areas have demonstrated successful induction of cortical plasticity through continuous cortical electrical stimulation (cCES) using subdural grids inserted at the time of surgical resection [59]. Another method of cortical stimulation is by way of rTMS. This is a non-invasive approach to target stimulation through serial sessions of theta-burst stimulation that, when applied to the motor cortex, can result in sustained depression and potentiation of cortical reactivity [60]. A similar approach has also shown promise in inducing functional changes in language motor areas in a patient with recurrent low-grade glioma [61]. However, despite the non-invasive nature of this intervention, there are limitations such as the need to undergo multiple sessions (ranging from 3–5 times per week), weak reported effects on sustained plasticity, and significant variability in response across patients [60]. Motor cortex stimulation (MCS) is yet another stimulation approach that has shown safety and efficacy in managing chronic neuropathic pain but has not been investigated in the context of tumors [62]. The surgical technique generally consists of placing a quadripolar electrode on the precentral gyrus, either perpendicular or parallel to the central sulcus. Pre-operative and intra-operative localization are used to verify placement over the motor cortex [62]. Due to the direct nature of this approach, MCS represents a promising tool for ‘pre-habilitation’ in patients with low-grade gliomas. A future surgical strategy may comprise the insertion of a stimulating electrode at the functional boundary of tumor resection in order to allow post-operative induction of plasticity. It is important to note that in these studies, stimulation methods were coupled with intensive motor or language rehabilitation as relevant to the tumor pathology, and this rehabilitation will likely be central to the success of these approaches. As such, stimulation in isolation may not be sufficient to consolidate plastic changes of motor tracts in the absence of the continued selective pressure imposed by volitional rehabilitation.

### 5.3. Metaplasticiy as a Marker of Plastic Potential

The next step along the evolutionary progression of integrating connectomics into the neurosurgical pipeline may involve the application of connectomics to metaplasticity, or the plasticity of plasticity, to compute an index of plastic potential at the individual level [41,44]. This index would comprise the potential of cerebral remapping and the subcortical constraints limiting this potential. Due to the inherent inter-individual variability in responsiveness to neuromodulation, such as with TMS, biomarkers of neuroplasticity would be particularly helpful when planning post-operative recovery strategies [63]. A better understanding of this concept has the potential to reshape our understanding of ‘eloquence’, whereby the eloquent brain may refer to neuroanatomical regions that represent important network hubs and have a low capacity for plasticity [41].

## 6. Limitations to Implementation

Collectively, the observations and points discussed herein demonstrate an existing basis for neurosurgeons to advance their knowledge of cortical networks to improve surgical strategies for glioma patients [16]. However, while connectome analysis is a feasible and novel approach to brain mapping in individual patients with brain tumors, it is not without its limits. Firstly, the approach of intricate pre-operative mapping can be time consuming and can stress some patients both pre-operatively and post-operatively. Careful selection of patients who may benefit from this strategy is essential and must consider patient illness severity, urgency of surgical intervention, as well as willingness and ability to participate. A patient-tailored approach is needed as the balance between functional and oncologic goals of surgery are individualized. For example, for symptomatic patients presenting with radiographic features suggesting high-grade glioma, time constraints or patient status may preclude the application of such sophisticated pre-operative studies. As such, this approach may lend itself most beneficially to patients with presumed low-grade gliomas as the relative immediacy of surgical intervention may not be present. This does not preclude the application of functional pre-surgical assessment, planning and application of connectomic concepts in malignant brain tumour surgery. Finally, the integration of connectomics into neurosurgical planning will undoubtedly be associated with greater healthcare costs for each patient, which may represent a challenge to its implementation in resource-limited settings. Cost-effectiveness assessment will be needed to determine how such an approach can be streamlined in an efficient and economical manner.

## 7. Future Directions

The intersection of functional neurosurgery and neuro-oncologic surgery represents an exciting frontier in the neurosciences. Integration of lessons learned from the disciplines of functional neurosurgery, epilepsy surgery, and neuro-oncology, along with advances in neuroimaging, neuropsychology paradigms, and technologies that allow us to modulate brain function non-invasively will revolutionize the surgical approach to intrinsic brain tumors such as high- and low-grade glioma. The traditional localizationist view of correlating brain function with specified cortical regions is not only outdated but also highly unreliable. Marked interindividual variability in anatomico-functional correlations exists and this variability occurs to an even higher degree in patients due to neuroplastic changes induced by tumors [64]. A network-based understanding can also inform epileptic outcomes in patients with gliomas. Identification of seizure onset zone using stereotactic EEG or MEG leading to analyses of neural eloquence, extent of resection, and disconnection necessary to achieve seizure freedom are indispensable tools in the armamentarium of a functional epilepsy neurosurgeon.

At present, a gold-standard approach for connectivity mapping does not exist, and the optimal mode remains to be determined [42]. There is limited evidence supporting the improved extent of resection or morbidity using pre-operative DTI, structural MRI, and fMRI. The goal of maximal safe resection based on these investigations alone is therefore inadequate in the complex surgical management of patients with glioma [13,65]. However, it should be noted that there may be challenges in ascertaining the accuracy of tractography in the setting of tumors where peri-tumoral edema or destruction of existing tracts may impact the data. Similarly, while intra-operative direct cortical stimulation has immense utility, it is usually constrained in identifying a focus of maximal activation for a given neurologic function [20]. Challenges in implementing connectivity-based surgical pipelines should not be underestimated, and ongoing coordinated research is needed to better resolve and define new ways to advance this field [42].

## 8. Conclusions

As has been demonstrated through the expanding applications within functional neurosurgery, personalized network-based approaches are anticipated to improve our overall understanding of brain function, as well as surgical, oncological, and functional outcomes for glioma patients. Moreover, there is a burgeoning field of peri-operative neuromodulation that can draw upon connectivity principles to rationalize target pathways to promote neuroplasticity and neurorehabilitation. The principles discussed herein may also be applied to other forms of non-invasive neuromodulation such as transcranial magnetic stimulation and MRI-guided focused ultrasound in the future. It remains to be determined how neuromodulation techniques can be incorporated within chemo–radiotherapy protocols, particularly for patients with high-grade glioma. Potentially, use of functional approaches for post-operative neurorehabilitation can be timed with oncological therapy. Coordinated research efforts are needed to aid in refining connectivity analyses derived through various methods and in designing and implementing trials to evaluate the promise that this emerging era of neuro-oncologic surgery holds.
